# Evaluating the Efficacy and Impact of Home-Based Cardiac Telerehabilitation on Health-Related Quality of Life (HRQOL) in Patients Undergoing Percutaneous Coronary Intervention (PCI): A Systematic Review

**DOI:** 10.3390/jcm14144971

**Published:** 2025-07-14

**Authors:** Francesco Limonti, Andrea Gigliotti, Luciano Cecere, Angelo Varvaro, Vincenzo Bosco, Rocco Mazzotta, Francesco Gravante, Nicola Ramacciati

**Affiliations:** 1 Department of Biomedicine and Prevention, University of Rome Tor Vergata, 00133 Rome, Italy; francesco.limonti@students.uniroma2.eu (F.L.); vincenzo.bosco@students.uniroma2.eu (V.B.); rocco.mazzotta@uniroma2.it (R.M.); 2 Department of Health and Exercise Science, The University of Oklahoma, Norman, OK 73019, USA; andrea92cv@gmail.com; 3 Department of Anesthesia and Resuscitation, A.O.R.N. Antonio Cardarelli, 80131 Naples, Italy; luciano.cecere@aocardarelli.it; 4 Department of Medicine and Cardiology, Provincial Healt Authority of Trapani, 91100 Trapani, Italy; angelo92vrv@gmail.com; 5 Department of Anesthesia, Local Health Authority of Caserta, 81031 Caserta, Italy; francesco.gravante@aslcaserta.it; 6 Department of Pharmacy, Health and Nutritional Sciences (DFSSN), University of Calabria, 87036 Rende, Italy

**Keywords:** telemedicine, self-management, patient compliance, remote monitoring, rehabilitation, patient-centered care

## Abstract

**Introduction:** Home-based cardiac telerehabilitation (HBCTR) is a multidisciplinary intervention aimed at optimizing functional, psychological, and social recovery in patients undergoing percutaneous coronary intervention (PCI). This rehabilitation model serves as an effective alternative to traditional center-based rehabilitation, providing a cost-effective and clinically advantageous approach. **Methods:** Following PRISMA guidelines, we conducted a systematic literature search across multiple databases (PubMed, CINAHL, Cochrane, Scopus, Web of Science). We included randomized controlled trials (RCTs), cohort, and observational studies assessing telerehabilitation in post-PCI patients. Primary outcomes focused on health-related quality of life (HRQoL) and adherence, while secondary outcomes included functional capacity (6 min walk test, VO_2_max), cardiovascular risk factor control, and psychological well-being. Risk of bias was assessed using the Cochrane RoB 2.0 and ROBINS-I tools. **Results:** A total of 3575 articles were identified after removing duplicates, of which 877 were selected based on title and abstract, and 17 met the inclusion criteria, with strong RCT representation ensuring robust evidence synthesis. HBCTR was associated with significant improvements in exercise capacity, with increases in VO_2_max ranging from +1.6 to +3.5 mL/kg/min and in 6 min walk distance from +34.7 to +116.6 m. HRQoL scores improved significantly, with physical and mental component scores increasing by +6.75 to +14.18 and +4.27 to +11.39 points, respectively. Adherence to telerehabilitation programs was consistently high, often exceeding 80%, and some studies reported reductions in hospital readmissions of up to 40%. Wearable devices and smartphone applications facilitated self-monitoring, enhancing adherence and reducing readmissions. Several studies also highlighted improvements in anxiety and depression scores ranging from 10% to 35%. **Conclusions:** HBCTR is a promising strategy for rehabilitation and quality-of-life improvement after PCI. It offers a patient-centered solution that leverages technology to enhance long-term outcomes. By integrating structured telerehabilitation programs, healthcare systems can expand accessibility, promote adherence, and improve equity in cardiovascular care.

## 1. Introduction

Cardiovascular diseases (CVD) represent one of the leading causes of morbidity and mortality worldwide, exerting a significant impact on both patients’ quality of life and healthcare systems [1]. In Europe, according to estimates from the World Health Organization (WHO), CVD accounted for 4.2 million deaths in 2019, corresponding to 42.5% of all-cause mortality [2]. Among the various forms of CVD, coronary artery disease (CAD) stands out as one of the most severe conditions, characterized by vascular lumen stenosis or occlusion and atherosclerotic spasms that may lead to myocardial ischemia or necrosis [3]. Despite advancements in primary and secondary prevention, CAD remains a major source of high morbidity and long-term disability in both industrialized and developing countries [4]. Over the past decades, percutaneous coronary intervention (PCI) has been introduced as a minimally invasive revascularization strategy, particularly for patients experiencing myocardial infarction (MI) [5]. Today, through technological advancements, PCI has become the primary treatment for CAD [6]. However, optimal postoperative recovery necessitates adequate cardiac rehabilitation (CR), a multidisciplinary intervention recommended by the WHO to optimize the physical, psychological, and social well-being of patients with CVD [7,8]. Substantial evidence demonstrated that CR improves health-related quality of life (HRQoL), reduces the risk of recurrent cardiac events, enhances functional capacity, and consequently lowers overall mortality rates [9]. Exercise training is a strong CR component, as improvements in functional capacity have been associated with a decreased risk of cardiovascular and all-cause mortality [10,11,12]. Despite its well-documented benefits and strong recommendations from organizations such as the American Heart Association and the American College of Cardiology [10,13], participation in CR programs remains remarkably low worldwide [14]. Only 10–30% of eligible patients enroll in center-based CR (CBCR) programs [10]. Key barriers include geographical constraints, organizational and economic limitations, and the lack of post-discharge care [15]. This underutilization of CR is even more pronounced among women, individuals living in rural areas, and ethnic minorities, highlighting persistent disparities in access to healthcare resources [16,17]. The recent COVID-19 pandemic has further underscored the urgent need for alternative strategies to provide remote rehabilitative care [18]. Concurrently, the development of new technologies, such as smartphones, wearable devices, and telemonitoring platforms (mHealth), has paved the way for home-based cardiac rehabilitation (HBCR or HBCTR), with preliminary evidence suggesting outcomes comparable to those of CBCR [19,20,21]. These digital solutions facilitate the delivery of rehabilitation prescriptions, adherence monitoring, and periodic assessments, effectively overcoming traditional barriers while reducing costs and travel time [22,23].

### Aim

This systematic review aims to synthesize the efficacy and impact of home-based cardiac telerehabilitation programs on HRQoL and clinical outcomes in patients who have undergone PCI. The primary objective is to identify the most promising strategies to enhance adherence, improve clinical outcomes, and provide an updated overview of key opportunities and challenges in implementing of HBCTR.

## 2. Materials and Methods

The systematic review was conducted from September 2024 to December 2024, according to the Preferred Reporting Items for Systematic Reviews and Meta-Analysis (PRISMA) guidelines [24,25]. The study protocol was registered in PROSPERO (No. CRD42024582933). The research protocol was published [26].

### 2.1. Eligibility Criteria

The eligibility criteria for this systematic review were defined using the PIO framework and established through collaborative consensus among the research team. Only primary empirical research studies published in peer-reviewed journals, written in English or Italian, and focusing on adult patients undergoing HBCTR after PCI were included.

To meet inclusion criteria, studies were required to report specific telerehabilitation interventions and measurable outcomes, such as HRQoL and therapeutic adherence. No restrictions on the year of publication were applied, ensuring comprehensive coverage of the literature. To preserve the review’s reliability and validity, non-empirical studies, secondary research, gray literature, and non-peer-reviewed articles were excluded. Additionally, studies published in languages other than English or Italian were omitted due to practical translation constraints. Research on pediatric populations, animal models, or pre-clinical experiments was excluded to maintain focus on the target adult population.

This review considered only primary studies, including randomized controlled trials (RCTs), cohort studies, cross-sectional studies, case-control studies, and qualitative research. Eligible studies had to involve patients undergoing remote cardiac rehabilitation post-PCI, include at least one quality of life assessment, and be published in English or Italian. All relevant studies available up to the extraction date were included in the review.

### 2.2. Information Sources

To identify potentially pertinent records, a comprehensive literature search was systematically conducted in the subsequent databases: PubMed (via MEDLINE), Scopus, CINAHL (via EBSCO), Cochrane Library (via Embase), and Web of Science (via EBSCO). The search was performed in November 2024.

### 2.3. Search Strategy

The search strategy was developed following the PIO (Population–Intervention–Outcome) framework (Table 1). The population (P) included adult patients who had undergone PCI and were enrolled in remote cardiac rehabilitation programs. The intervention (I) encompassed the use of telemedicine, telerehabilitation, and telenursing programs, while the outcomes (O) focused on HRQoL, adherence rates, and the reduction of clinical events.

The comparison (C) component was not included, as it was outside the scope of this review. To construct the search strings, keywords, controlled vocabulary (e.g., MeSH terms), and Boolean operators (AND/OR) were used and customized for each database. These terms were selected based on the eligibility criteria to maximize coverage. No search filters were applied to avoid narrowing the results. In line with established methodological guidelines, the full search strategy for PubMed and the finalized search strings for all databases were documented to ensure transparency and reproducibility.

### 2.4. Data Selection, Collection Process, and Data Items

The study selection process consisted of two phases: a first screening of titles and abstracts, followed by an evaluation of the full text. All identified potential references were imported into Rayyan©, an online platform used to improve the efficiency and transparency of the screening process [27]. In the initial phase, titles and abstracts were compared with the established eligibility criteria. For the eligible studies, the full text was obtained through Zotero© v7.0, online searches, and institutional sources and read for a final assessment on the inclusion and exclusion criteria. Two reviewers (A.V. and L.C.) independently extracted the information, addressing discrepancies through structured comparisons with the third researcher (F.L.). The data collected included author, year, country, population, inclusion criteria, diagnosis, setting, intervention, duration, primary and secondary outcomes, results, CCAT (Crowe Critical Appraisal Tool) score [28], and level of evidence. The included studies were classified based on their level of evidence (Table 2) according to the Polit and Beck hierarchy [29,30]. Subsequently, the results were summarized in a synoptic table that provided a comprehensive synthesis of the evidence and explored relationships within and between studies to identify emerging patterns.

### 2.5. Quality Assessment and Risk of Bias

The risk of bias was assessed using the CCAT [31], a tool designed to assess the methodological quality of studies across a variety of designs. This structured approach ensures a detailed and robust examination of the studies included in the review. Each study was independently reviewed by two reviewers (L.C. and A.V.), and any discrepancies were resolved by a third reviewer (F.G.) to ensure objectivity and consensus. The CCAT scoring system assigns scores from 0 (poor quality) to 5 (excellent quality) for each category, based on the overall assessment of eight domains, with an overall score determined by the sum of the assessments of all sections and expressed as a percentage.

To provide a structured, domain-specific assessment of potential biases, particularly relevant for subjective outcomes such as psychological well-being, adherence, and self-reported quality of life, we applied risk of bias assessment tools tailored to the study design. Additionally, the risk of bias was assessed using the Polit and Beck framework [29], which provided a complementary analysis of critical dimensions, including sample selection, internal and external validity, and the appropriateness of the study design. Specifically, the RoB 2 tool was used for RCTs (Table 3) and the ROBINS-I tool was applied to non-randomized studies (Table 4) [32,33]. These tools allowed for a thorough assessment of key domains of bias, including the randomization process, deviations from intended interventions, missing outcome data, outcome measurement, and selective reporting.

This assessment strategy is adopted to strengthen our narrative synthesis. Given the heterogeneity in study designs, interventions, and outcomes, a meta-analysis was not performed. Instead, the results were qualitatively synthesized to identify recurring patterns, emerging themes, and critical methodological limitations across the studies.

### 2.6. Effect Measures

Following the PRISMA guidelines [24], the synthesis of results was based on the data extracted from the included studies, which assessed the relationship between the rehabilitation intervention and improvement in quality of life. To maintain the integrity of the original data, statistical significance was reported as presented in the individual studies without standardization between studies. This approach ensured a consistent and accurate representation of the evidence relevant to the systematic review’s objectives.

### 2.7. Synthesis Methods

Methodological variability and different measures in the studies included in this review prevented an aggregate quantitative synthesis, as outlined in the latest edition of the Cochrane Handbook for Systematic Reviews of Interventions [51].

Although most studies reported improvements in HRQoL and functional capacity, potential sources of bias were identified using the RoB 2 and ROBINS-I instruments. Heterogeneity in outcome measurement tools, intervention modalities, and analytic approaches contributed to limited methodological and statistical consistency.

A narrative synthesis was therefore adopted. For each included study, key details regarding the study design, population, interventions, and primary outcomes were summarized. Results were stratified by intervention type and duration to allow comparisons across comparable subgroups.

Primary outcomes included HRQoL and adherence to drug therapy, while secondary outcomes included functional capacity (e.g., 6 min walk test, VO_2_max), control of cardiovascular risk factors, incidence of major adverse cardiovascular events, and psychological well-being.

## 3. Results

### 3.1. Study Selection

**Figure 1:**
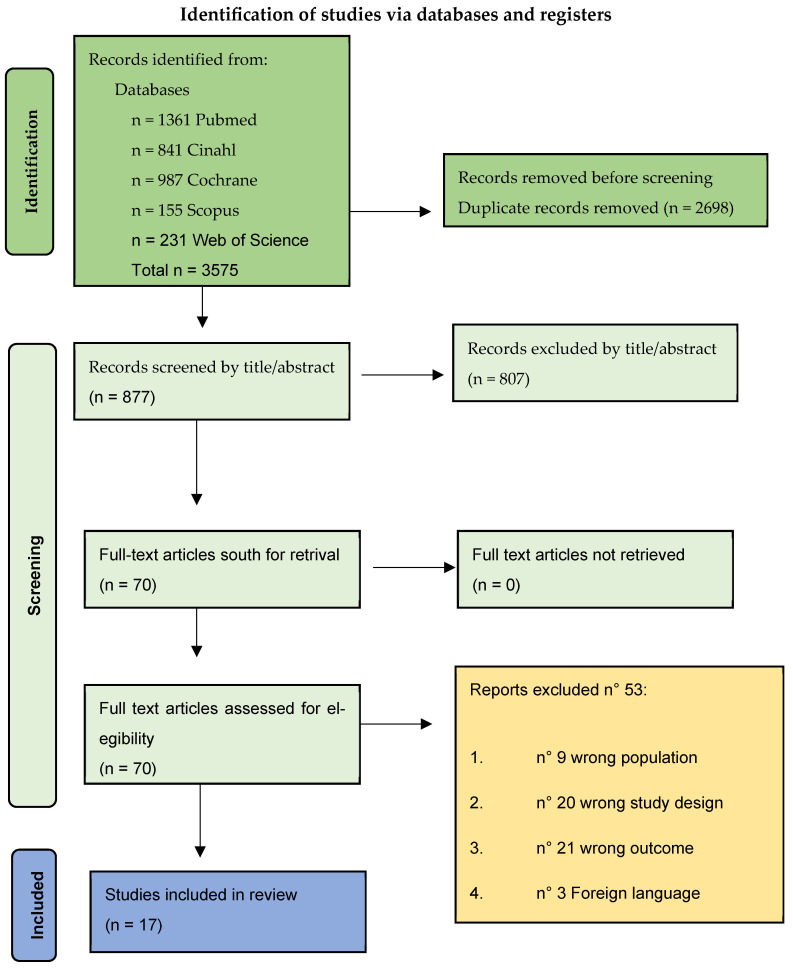
PRISMA flowchart of selected studies.

The initial search of five electronic databases identified *n* = 3575 potentially relevant articles. After removing duplicates (*n* = 2698), the remaining records (*n* = 877) were screened based on their titles and abstracts. After this screening phase, the records deemed relevant were passed to full-text assessment (*n* = 70). During this phase, 53 articles were excluded. This resulted in 17 articles meeting the inclusion criteria for this review, as detailed in the PRISMA diagram [24] (Figure 1).

### 3.2. Study Characteristics and Interventions

Most of the included studies employed a randomized design (RCTs) where telerehabilitation protocols were implemented at home [34,35,37,39,40,41,42,43,44,49]. Other studies included observational cohort studies [46], retrospective comparative studies [47], cross-sectional and crossover studies [50], and prospective cohort studies [48,49]. Participants, both male and female, were typically aged 50–75 years and had undergone PCI for stable acute or chronic coronary syndrome, often with hypertension and controlled diabetes. Only clinically stable patients, without arrhythmias, severe heart failure, or significant ischemia, were included, while those with kidney failure, lung disease, or dementia required supervision for home-based exercises. The duration of interventions ranged from 8 weeks to 12 months, with follow-ups at 3 or 6 months [39,48]. Studies were conducted in China, Europe, Australia, and the USA, frequently utilizing mobile applications such as WeChat, HeartLab, and EVITE [43,49,50] as well as wearable devices (e.g., Polar H10, Fitbit, Xiaomi Mi Band) for monitoring physical activity [44,48]. These tools enabled structured aerobic exercise training with progressive intensity [37,40], alongside education on diet, stress management, and treatment adherence [47,48]. Clinical parameters such as VO_2_max, MET, and quality of life were assessed using validated tools [39,44].

### 3.3. Study Quality Assessment and Level of Evidence

In the systematic review, the quality assessment of the studies reported a score between 72.5% and 90% (Table 5). The analyzed studies, including trial articles, case studies, and clinical documents, are categorized as Levels I to VI of evidence, as detailed in Table 2. Specifically, there are eleven Level II studies [34,35,37,38,39,40,41,42,43,44,49,52], two Level III studies [45,50], and four Level V studies [46,47,48,49] (Table 5). This classification highlights a gradual increase in the level of evidence of studies on HBCTR, while also underscoring the observational and descriptive nature of the data, which, although valuable, reflect a lower hierarchy of clinical evidence compared to randomized controlled trials or meta-analyses.

### 3.4. Record Included for Review

The final selection comprised 17 studies that met the inclusion criteria and were included in this systematic review. These studies varied in terms of design, population characteristics, intervention modalities, and outcome measures. Table 6 provides a comprehensive overview of each study, detailing authorship, publication year and country, study design, sample size, participant demographics, inclusion criteria, underlying diagnoses, intervention characteristics, duration, primary and secondary outcomes, and main findings.

### 3.5. The Role of Wearable Devices

The integration of wearable devices in cardiac rehabilitation has significantly enhanced personalized treatment and continuous monitoring, leading to improved physical capacity and quality of life. Studies have demonstrated that telerehabilitation programs incorporating wearable technology, such as smart bands and ECG monitoring systems, facilitate structured aerobic exercises, resulting in notable increases in the six-minute walk test (6MWT), maximal oxygen consumption (VO_2_max), and metabolic equivalents (METs) after interventions lasting between 6 weeks and 12 months [35,37,39]. Programs utilizing real-time monitoring and progressive exercise intensity have shown greater improvements in exercise capacity compared to standard rehabilitation approaches. High-intensity interval training (HIIT) appears particularly beneficial in enhancing muscle strength and pulmonary function with VO_2_max increments of up to 12% and muscle strength gains of approximately 10%, while moderate-intensity continuous training (MICT) remains effective in improving overall endurance [37]. Additionally, mobile applications and wearable devices have enabled remote monitoring, leading to a 15–20% improvement in mental health, reductions in anxiety and depression, and an increase in treatment adherence [43,48]. Beyond physical and psychological benefits, telerehabilitation has been associated with a reduction in family burden and a 20% decrease in rehospitalization rates, emphasizing its role in long-term disease management [42]. Studies conducted during the COVID-19 pandemic further highlighted the effectiveness of remote rehabilitation in maintaining continuity of care, ensuring that patients remained engaged in structured exercise programs despite logistical barriers [48].

### 3.6. Using the Web and Smartphone Apps

The integration of mobile applications in home-based cardiac telerehabilitation (HBCTR) has significantly improved patient adherence, clinical outcomes, and lifestyle modifications. These apps enable personalized exercise prescriptions, real-time monitoring, and interactive feedback, leading to measurable gains in physical and psychological well-being. WeChat-based programs in China have facilitated remote supervision through educational materials and digital progress tracking, resulting in a 15% improvement in mental health, an increase in VO_2_max (+1.2 mL/kg/min), and a reduction in LDL levels (−10 mg/dL) [46,49]. Similarly, EVITE, integrating self-assessment and lifestyle coaching, led to a 30% increase in weekly physical activity, an LDL reduction (−8.2 mg/dL, *p* < 0.04), and improved quality of life (+4.4 SF-12 points); 75% of users also quit smoking [43]. Gamification-based interventions have further enhanced adherence. HeartHab improved exercise compliance and reduced anxiety and depression [50]. A 12-week app-based intervention [34] incorporating medication reminders and personalized physical activity guidance led to a 50 m increase in 6MWT, a 12-point SF-36 quality of life improvement, and reductions in blood pressure and cholesterol (*p* < 0.05). In Australia, the SMART-REHAB program improved 6MWT performance (116.6 vs. 91.4 m, *p* = 0.02) and adherence to therapy [40]. Similarly, the Cardioplan app combined physical activity tracking with heart rate monitoring, leading to improved aerobic capacity (VO_2_max: +1.62 mL/kg/min), better diet adherence, and reduced psychological stress [44].

### 3.7. Remote Home Training Program

Home-based cardiac telerehabilitation (HBCTR) has proven effective in enhancing post-PCI recovery by integrating remote monitoring, guided exercise, and motivational support. Li et al. [38] demonstrated that a six-month HBCTR program improved exercise capacity (increased 6MWT), enhanced medication adherence, and reduced adverse cardiovascular events. In Italy, elderly post-acute coronary syndrome patients followed a hybrid rehabilitation model, combining in-center sessions with a home-based program including resistance training and progressive walking. At six months and one year follow-ups, handgrip strength improved (+10 kg), gait speed increased (+0.18 m/s), and anxiety and depression levels decreased [41]. These improvements were correlated with the reduced mortality and cardiovascular rehospitalization. The integration of supervised exercise with remote monitoring optimizes functional capacity and long-term health outcomes, reinforcing the role of HBCTR as a scalable and effective post-PCI rehabilitation strategy.

## 4. Discussion

In recent years, interest in HBCTR has grown significantly. Supported by remote monitoring and surveillance tools, this approach has the potential to substantially improve the quality of life of patients with coronary artery disease, offering an effective alternative to CBCR. The primary outcome of this systematic review was to analyze the effects of cardiac telerehabilitation on HRQoL in patients undergoing PCI. We also investigated which technologies were used and whether they helped patients improving adherence to telerehabilitation programs, as well as reduce the incidence of recurrent cardiac events. However, a critical issue that emerged from the review is the limited availability of long-term follow-up data. While most studies have focused on short-term improvements in health-related quality of life (HRQoL), exercise capacity, and psychological well-being, only a few have extended beyond 6 or 12 months or assessed clinical endpoints such as hospital admission or major adverse cardiovascular events. For example, the Chinese HBCTR study followed patients for up to 48 months and reported a significant reduction in new cardiac events and improved adherence [46]. Another study conducted in an older population following myocardial infarction observed that improvements in health-related quality of life were not only significant in the short term but also maintained up to one year after the intervention [41].

Several studies have shown that patients participating in HBCTR programs experience significant improvements in exercise capacity, symptom reduction, and perceived psychological well-being. In particular, programs integrating continuous physiological monitoring, such as heart rate and oxygen saturation, provide added value in terms of safety and adherence to therapy [35]. Remote monitoring also allows healthcare workers to provide real-time feedback, improve patient motivation, and ensure ongoing follow-up, often comparable to in-person rehabilitation [44]. However, the lack of direct supervision during exercise sessions may lead to lower intensity or frequency of the rehabilitation sessions, reducing their overall effectiveness [36]. From a psychological perspective, HBCTR has been shown to significantly reduce levels of anxiety and depression [47]. In particular, programs incorporating educational components and psychological support, delivered through telemedicine tools or virtual meetings, appear to have a superior impact compared to standard rehabilitation models [53]. Remote psychological interventions integrated into HBCTR programs contribute to long-term adherence by promoting self-efficacy and behavioral change [22]. Additionally, some objective physiological parameters were used to assess the efficacy of HBCTR; for example, significant increases in VO_2_max, ranging from +1.6 to +3.5 mL/kg/min [37] and gains of up to +116 m in the 6MWT [48] were reported following home-based interventions, indicating measurable improvements in cardiopulmonary fitness.

Left ventricular ejection fraction (LVEF) also showed modest but consistent improvement post-intervention [47]. Biochemical indicators such as lipid profiles (e.g., LDL reduction), HbA1c, and blood pressure were monitored during the HBCTR periods and reported positive results [38,46,50]. These results reinforce the utility of HBCTR not only in improving perceived quality of life but also in restoring physical function.

Regular interaction with healthcare professionals, even in a virtual setting, helps reinforce motivation and adherence to therapeutic prescriptions, reducing the risk of dropout and improving continuity of care. In this context, the role of nurses is crucial, not only in remote monitoring of clinical parameters, but also in providing educational and motivational support to patients. Several studies have highlighted how nursing interventions in HBCTR improve program adherence, reduce the risk of adverse events, and promote lasting lifestyle changes [34,45]. The opportunity for personalized interaction with the patient, through motivational coaching and continuous support, increases self-efficacy and contributes to greater engagement in the rehabilitation programs [40]. The flexibility offered by home-based rehabilitation can increase participation by reducing logistical barriers associated with center-based programs. However, ensuring an adequate level of personalization and monitoring is essential to prevent patients from following the rehabilitation protocol less rigorously [36]. Although higher levels of adherence and satisfaction have been frequently reported in HBCTR interventions, behavioral factors such as scheduling flexibility, greater autonomy, ease of use of digital platforms, perceived safety through continuous remote monitoring, and integration of psychological support have not been explored. However, they are of fundamental importance to ensure and contribute to long-lasting engagement. Some studies have suggested that a hybrid approach, combining supervised in-center sessions with remote support for home-based management, could be the optimal solution to maximize the benefits of cardiac rehabilitation [44]. Despite the numerous advantages of HBCTR, certain barriers to its large-scale implementation persist. Digital literacy, particularly among elderly patients, represents a significant obstacle to the use of telemonitoring platforms and remote rehabilitation management applications [45]. Furthermore, patient acceptance of a non-traditional rehabilitation model may vary based on socio-cultural factors and familiarity with digital technologies [54]. Some patients may prefer direct interaction with the healthcare team, believing that the hospital setting provides a greater sense of security and control.

## 5. Limitations

The included studies demonstrated substantial heterogeneity in the HBCTR protocols, with variations in duration, frequency, and intensity of exercises, as well as in the monitoring tools and digital platforms used. This variability limits the generalizability of the findings and hinders the identification of a universally optimal telerehabilitation model for PCI patients. Furthermore, only a few studies addressed key aspects such as operational feasibility, cost effectiveness, and scalability factors that are essential for real-world implementation, particularly in resource-limited settings or among patients with low digital literacy. Around 75% of the RCTs included were conducted in China, which raises questions about the external validity of the results. Healthcare delivery models, digital infrastructure, and patient engagement strategies in the Chinese context may differ significantly from those in Western countries, such as European or North American countries. Therefore, it is necessary to carry out new research in different clinical, cultural, and organizational contexts to generalize the results on HBCTR. Another important limitation concerns the lack of long-term follow-up in most studies, making it difficult to assess the durability of the observed benefits, especially regarding quality of life, psychological well-being, and adherence to treatment. Future research should aim to develop more standardized and adaptable protocols tailored to diverse patient profiles, supported by economic evaluations that confirm their sustainability. The adoption of mixed-method approaches in future studies, including patient-reported experience measures, could help better capture and understand the behavioral and contextual dimensions influencing engagement and satisfaction elements that are essential to optimize the design, effectiveness, and reproducibility of HBCTR programs. Hybrid models that combine initial in-person assessment with remote follow-up may help improve safety and personalization. Additionally, longer follow-up studies and targeted strategies to improve digital accessibility and inclusiveness of rehabilitation programs are needed to ensure greater and more equitable access. Moreover, future studies should further explore the impact of HBCTR not only on physical and functional outcomes, but also on biochemical and physiological markers and inflammatory parameters. The integration of such endpoints could offer a more comprehensive evaluation of cardiovascular risks and the effectiveness of telerehabilitation beyond exercise capacity and quality of life.

## 6. Conclusions and Future Directions

This systematic review highlights the promising and safe nature of HBCTR in improving HRQOL among patients undergoing PCI. The use of telemonitoring resources, such as smartphone applications, wearable devices, and web-based platforms, facilitates optimal adherence to rehabilitation programs, mitigates the risk of clinical relapse, and allows for timely intervention in response to warning signals. Furthermore, real-time feedback mechanisms enhance patient motivation, ensure continuity of care, and promote a more active and health-oriented lifestyle. The results of the included studies indicate that structured and remotely supervised home-based exercise regimens improve key indicators, including VO_2_max, six-minute walk test (6MWT), and metabolic equivalents (METs). Simultaneously, a psychophysiological benefit emerges, exemplified by reductions in anxiety and depression, particularly when the intervention includes health education, psychological support, and motivational strategies. However, variability in rehabilitation protocols and the paucity of extensive follow-up data warrant caution in interpreting these results, highlighting the need for further research to develop standardized guidelines on exercise duration and intensity. In conclusion, HBCTR appears to be an effective care model that can improve functional recovery and overall quality of life after PCI. Although technological and cultural barriers remain, large-scale implementation of HBCTR initiatives offers a real opportunity to improve both accessibility and equity in cardiac care. Future research should explore the integration of advanced digital health technologies, such as artificial intelligence and predictive analytics, into HBCTR pathways. Additionally, further studies are needed to assess real-world adaptability, cost-effectiveness, and strategies to reduce digital exclusion, especially among older adults and vulnerable populations.

## Figures and Tables

**Table 1:** PIO framework.

Categories	Description
Population (P)	The population included in this review was composed of individuals ≥ 18 years of age who underwent PCI after MI.
Intervention (I)	The intervention included programs of cardiac rehabilitation conducted remotely via telemedicine or telenursing. These programs aim to provide higher flexibility, individualization, and better adherence to the rehabilitation process, as they allow the patients to comfortably follow the sessions from their own homes.
Outcome (O)	The primary outcome of this review was health-related quality of life (HRQoL) post-rehabilitation, measured using validated tools reported in the included studies. Studies were considered eligible if HRQoL was assessed quantitatively through established measurement instruments, such as SF-36, EQ-5D, or other validated scales. The included interventions involved home-based telemedicine programs. The use of devices such as phones, tablets, or computers is required to engage in the rehabilitation process.

PCI: percutaneous coronary intervention; MI: myocardial infarction; HRQoL: health-related quality of life; SF-36: 36-item short from survey; EQ-5D: EuroQuol-5D.

**Table 2:** Type of method and level of evidence of the studies included.

Level of Evidence	Definition
Level I	From a systematic review/meta-analysis of all relevant random control trials (RCT’s)
Level II	Obtained from well-designed RCT’s
Level III	Obtained from well-designed trials without randomization (quasi-experiment)
Level IV	From well-designed cohort or case control studies
Level V	From systematic reviews of descriptive or qualitative studies
Level VI	From single qualitative or descriptive studies
Level VII	From expert opinion/committees

Proposed by Polit and Beck (2017) [29] and adapted from Johanns et al. (2017) [30] p. 91.

**Table 3:** Risk of bias assessment (RoB2).

Study	Randomization Process	Deviations from Intended Interventions	Missing Outcome Data	Bias in Measurement of the Outcome	Selection of the Reported Result	Overall Bias
Lao et al. [34]	Low	Low	Low	Some concerns	Low	Some concerns
Lee et al. [35]	High	High	Low	High	High	High
Chen W. et al. [36]	Some concerns	Low	Low	Low	Low	Low
Yakut et al. [37]	Some concerns	Low	Low	Some concerns	Low	Some concerns
Li et al. [38]	Low	Low	Low	Some concerns	Low	Low
Fang et al. [39]	Low	Low	Low	Some concerns	Low	Low
Yudi et al. [40]	Low	Low	Low	Low	Low	Low
Campo et al. [41]	Low	Low	Some concerns	Low	Low	Some concerns
Zheng et al. [42]	Low	Low	Low	Some concerns	Low	Low
Bernal-Jiménez et al. [43]	Some concerns	Low	Low	Some concerns	Low	Some concerns
Dalli Peydro et al. [44]	Low	Low	Low	Low	Low	Low

**Table 4:** Risk of bias in non-randomized studies of interventions (ROBINS-I).

Study	Confounding	Selection of Participants	Classification of Interventions	Deviations from Intended Interventions	Missing Data	Measurement of Outcomes	Selection of the Reported Result	Risk of Bias
Calvo-López et al. [45]	Moderate	Low	Low	Low	Low	Moderate	Low	Moderate
Ma et al. [46]	Serious	Moderate	Moderate	Moderate	Moderate	Moderate	Moderate	Serious
Yu et al. [47]	Moderate	Low	Moderate	Low	Low	Moderate	Low	Moderate
Gu et al. [48]	Moderate	Low	Low	Low	Low	Moderate	Moderate	Moderate
Chen et al. [49]	Serious	Moderate	Serious	Serious	Moderate	High	Serious	Serious
Sankaran et al. [50]	Moderate	Low	Low	Low	Low	Low	Low	Low

**Table 5:** Type of study and level of evidence.

N	Country	Reference	1	2	3	4	5	6	7	8	Total	%	Type of Study	LoE
1	Spain	Dalli Peydró, E. et al. [44]	4	4	2	3	4	4	4	4	29	72.5%	Randomized controlled trial	II
2	China	Chen, W. et al. [36]	4	4	3	4	3	4	4	4	30	75%	Randomized controlled trial	II
3	Spain	Bernal-Jiménez, M. et al. [43]	5	4	3	4	3	4	5	4	32	80%	Randomized controlled trial	II
4	New Zealand	Yudi, M. B. et al. [40]	4	3	4	4	3	3	3	4	28	70%	Randomized controlled trial	II
5	China	Zheng, Y. et al. [42]	5	4	4	3	4	3	3	4	30	75%	Randomized controlled trial	II
6	Spain	Calvo-López, M. et al. [45]	4	3	3	2	4	3	3	4	26	65%	Cross sectional study	III
7	China	Ma, J. et al. [46]	4	3	4	3	4	3	3	4	28	70%	Observational cohort study	V
8	China	Yu, H. et al. [47]	4	4	3	4	4	4	3	4	30	75%	Retrospective comparative study	V
9	Turkey	Yakut, H. et al. [37]	4	4	3	4	4	3	4	3	29	72.5%	Randomized controlled trial	II
10	China	Li, Z. et al. [38]	4	3	4	4	3	4	3	4	29	72.5%	Randomized controlled trial	III
11	Belgium	Sankaran, S. et al. [50]	4	5	4	4	4	4	3	4	32	80%	Multidisciplinary crossover study	III
12	Korea	Lee, Y. H. et al. [35]	4	4	5	4	4	4	3	4	32	80%	Randomized controlled trial	II
13	China	Gu, J. et al. [48]	4	4	3	4	4	4	3	5	32	80%	Prospective cohort study	V
14	China	Fang, J. et al. [39]	4	3	4	3	3	4	4	4	30	75%	Randomized controlled trial	II
15	Italy	Campo, G. et al. [41]	5	4	5	4	5	4	4	5	36	90%	Randomized controlled trial	II
16	China	Lao, S. S. W. et al. [34]	4	5	4	4	5	4	5	4	34	85%	Randomized controlled trial	II
17	China	Chen et al. [49]	4	4	3	5	4	4	4	4	32	80%	Prospective cohort study	V

**Table 6:** Table of included studies.

N°	Author(s)	Year and Country of Study	Type of Study	Inclusion Criteria	Intervention(s) Type/s and Duration	Outcome(s) and Results	CCAT
1	Ernesto Dalli Peydro, [44]	Spain, 2022	RCT	N = 67 patients; low-risk acute coronary syndrome patients, aged 18–72 years, left ventricular ejection fraction ≥ 50%, minimum smartphone usage skills	Comparison of a 10-month cardiac telerehabilitation (CTR) program with a conventional 8-week center-based cardiac rehabilitation (CBCR) program. Duration: CTR 10 months and CBCR 8 weeks	Primary Outcome: Increased physical activity (measured in MET min/week using IPAQ). Secondary Outcomes: VO_2_max, adherence to a Mediterranean diet, psychological well-being, health-related quality of life, and smoking cessation; CTR group showed greater increases in physical activity, VO_2_max, and adherence to a Mediterranean diet; The program also reduces dropouts and favors the return to work. Psychological distress and quality of life were better in the CTR group compared to the CBCR group.	29
2	Wanping Chen, [49]	China 2024	RCT	N = 106 patients (47 in the home-based group, 50 in the center-guided home-based group); patients aged 35–70 years. Confirmed diagnosis of acute coronary syndrome (ACS). Completed coronary revascularization. Stable condition post-revascularization. No severe comorbidities or contraindications for exercise	Group A: Home-based rehabilitation. Group B: Center-guided home-based rehabilitation. Both groups underwent personalized exercise prescriptions and cardiopulmonary exercise testing (CPET) before and after 12 weeks of rehabilitation. Duration: 12 weeks	Primary Outcome: Peak oxygen uptake (VO_2_peak). Secondary Outcomes: Maximum metabolic equivalents (METs), anaerobic threshold oxygen uptake (VO_2_AT), maximal workload (Load max), cardiopulmonary reserve capacity; Significant improvements in METs, VO_2_peak, Load AT, and VO_2_AT in the center-guided home-based group compared to the home-based group. The center-guided group achieved better outcomes in a shorter time, highlighting the importance of supervised rehabilitation	30
3	Bernal-Jiméne, [43]	Spain 2024	RCT	N = 128 patients, 67 in the mHealth group and 61 in the control group; the study included patients with CAD aged between 18 and 75 years who owned a smartphone with an internet connection during the whole study period that have received percutaneous coronary intervention (PCI) with stent implantation.	mHealth Group: Patients used the EVITE app, which provided self-monitoring tools, personalized feedback, educational content, and motivation strategies to support lifestyle changes. Control Group: Received standard healthcare with verbal and written recommendations on lifestyle changes. Duration: 9 months	Primary Outcomes: Adherence to the Mediterranean diet, frequency of healthy food consumption, physical activity (self-reported), smoking cessation, knowledge of healthy lifestyle, quality of life, therapeutic adherence, and overall satisfaction. Secondary Outcomes: BMI, waist circumference, blood pressure (systolic and diastolic), heart rate, HbA1c, lipid profile, anxiety and depression, and major adverse cardiovascular events (MACE); The mHealth group showed significant improvements compared to the control group: Mediterranean diet adherence: 11.83 vs. 10.14 points (*p* < 0.001). Physical activity: 619.14 vs. 471.70 min/week (*p* = 0.007). Smoking cessation: 75% vs. 42% of smokers stopped (*p* = 0.01). Quality of life: Higher physical component score (45.80 vs. 41.40, *p* = 0.02). Therapeutic adherence: Comparable between groups (84% vs. 78%). No significant differences in BMI, blood pressure, or lipid profile. Anxiety and depression decreased in both groups	32
4	Yudi, [40]	Australia 2020	RCT	N = 206 patients with acute coronary syndromes (ACS), of whom, 168 completed the follow-up; Adults aged 18 years or older. Diagnosis of ACS with documented coronary artery disease (≥50% stenosis). Treated with medical therapy or percutaneous coronary intervention (PCI). Ownership of a smartphone. No significant exercise limitations or life expectancy < 1 year.	Smartphone-based Cardiac Rehabilitation Program (S-CRP): Patients received guidance on exercise, medication adherence, and lifestyle changes through a smartphone app. Usual Care (UC): Patients received traditional rehabilitation referral. Duration: 8 weeks	Primary Outcome: Change in exercise capacity (6 min walk test distance). Secondary Outcomes: Adherence to cardiac rehabilitation, changes in cardiac risk factors, psychological well-being (anxiety and depression), and quality of life (SF-36 and EQ5D); of the 168 patients with complete follow-up. At 8-week follow-up, the S-CRP group had a clinically significant improvement in 6 min walk test distance. Higher adherence and uptake of rehabilitation in the S-CRP group (adherence: 72% vs. 22%, *p* < 0.001). No significant differences in cardiac risk factors, anxiety, or depression between groups. Comparable quality of life improvements in both groups	28
5	Zheng Y., [42]	China 2024	Randomized controlled trial (RCT)	N = 106 patients (53 in the intervention group, 53 in the control group). Patients had undergone percutaneous coronary intervention (PCI).	Intervention Group: Received routine rehabilitation care and home-based cardiac telerehabilitation with monitoring and feedback. Control Group: Received routine care without additional telemonitoring. Duration: 3 months	Primary Outcomes: Improvements in exercise endurance (6 min walk test, VO_2_max), and cardiac function (left ventricular ejection fraction, anaerobic threshold). Secondary Outcomes: Quality of life (measured with the Short-Form 12 scale), mental and physical component summary scores, and family burden status. Significant improvements in the intervention group compared to the control group in: 6 min walk test: Increased distance covered. VO_2_max and anaerobic threshold: Significant improvement in exercise capacity. Quality of life: Higher scores on physical and mental health components (Short-Form 12). Family burden status: Reduction in perceived family burden due to illness	30
6	Margarita Calvo-López, [45]	Spain 2023	Cross-sectional Pilot Study	N = 50 patients completed the program. Patients who experienced an acute myocardial infarction (AMI) in the previous 3 months. Left ventricular ejection fraction ≥ 40%. Access to a mobile device or tablet. Did not participate in any previous cardiac rehabilitation program	Holistic home-based cardiac telerehabilitation program (telematic CRP) including: Tailored aerobic and strength training (3 weekly sessions). Educational sessions on lifestyle habits, therapeutic adherence, and patient empowerment (2 weekly sessions). Delivered via the HumanITcare telemedicine platform.	Primary Outcomes: Improvements in functional capacity and muscle strength. Secondary Outcomes: Adherence to the Mediterranean diet, emotional state (anxiety and depression), quality of life (EuroQoL questionnaire), and program feasibility and safety; Significant improvements in functional capacity (+1.6 METs), muscle strength (arm curl +15.5%, sit-to-stand +19.7%), weekly training volume (+803 METs), adherence to the Mediterranean diet, and anxiety levels. No major complications occurred; adherence was >80% for both exercise and educational sessions.	26
7	Ma J., [46]	China 2021	Prospective Observational Cohort Study	N = 335 patients. 170 in home-based cardiac rehabilitation (HBRC) group, 165 in control group. Age ≥ 18 years. Successful PCI (residual stenosis < 30% without complications). Smartphone ownership with an active WeChat account.	HBCR Group: Patients received weekly health education materials and monthly personalized exercise prescriptions via a WeChat-based smartphone platform. Interaction included telemonitoring, telecommunication, and real-time data sharing. Control Group: Received standard care, including a one-time 20 min health education session. Duration: 42 months	Primary Outcome: Incidence of major adverse cardiac events, including cardiovascular death, non-fatal myocardial infarction, unscheduled coronary revascularization, and non-fatal stroke. Secondary Outcomes: Exercise capacity (measured in METs). Quality of life (Seattle Angina Questionnaire). Psychological status (GAD-7 and PHQ-9 scores). Risk factor management and unscheduled hospitalizations; HBCR group demonstrated significantly lower major adverse cardiac events incidence (1.5% vs. 8.9%; *p* = 0.002). Improved exercise capacity (mean METs: 6.2 vs. 5.1; *p* = 0.002). Better quality of life scores and superior risk factor control compared to the control group. Reduced unscheduled readmissions (9.7% vs. 23%; *p* = 0.002)	28
8	Yu H., [47]	China 2021	Retrospective comparative study	N = 115 patients 57 in Group A 58 in Group B; Patients with acute myocardial infarction (AMI) confirmed by emergency coronary angiography. Successful PCI without contraindications. Clear consciousness and no communication barriers.	Group A (Control): Routine rehabilitation guidance including health education, diet, and medication adherence. Group B (Intervention): Early home-based cardiac rehabilitation (CR) exercise including personalized plans with aerobic exercises during hospitalization and after discharge. Duration: 3 months	Improved ejection fraction, systolic and ventricular function. 6 min walking distance (6MWD). Improvement of cardiac antioxidant index). Exercise endurance (exercise duration, anaerobic threshold, VO_2_). Improved quality of life; early home-based CR exercise improved cardiac function. Longer 6MWD (458.96 m vs. 352.12 m *p* < 0.05). Higher cardiac antioxidant levels. Increased exercise endurance with increase of VO_2_max. Better quality of live across multiple dimensions.	30
9	Yakut H., [37]	Turkey, 2022	RCT	N = 21 patients (11 in the HIIT group, 10 in the MICT group); The patients with myocardial infarction, 35–65 years, left ventricular ejection fraction > 50%, clinically stable and able to walk independently.	Home-based HIIT group with high-intensity interval training performed twice a week, focusing on intervals of 85–95% heart rate reserve. MICT Group: Moderate-intensity continuous training performed twice a week, focusing on 70–75% heart rate reserve. The programs included warm-up, main exercise, and cool-down sessions. Duration: 12 weeks	The primary outcome measure was functional capacity using 6 min walking test (6MWT). Secondary outcomes included resting blood pressure and HR, peripheral oxygen saturation, pulmonary function and respiratory muscle strength, dyspnea severity, body composition, peripheral muscle strength, and health-related quality of life (HRQoL). Both HIIT and MICT groups showed significant improvements in functional capacity, pulmonary function, and quality of life (HRQoL). HIIT was more effective than MICT in improving lower limb muscle strength (e.g., knee extensor strength) and pulmonary function. Significant decreases in BMI and body fat percentage were observed in both groups, though differences between groups were not statistically significant.	29
10	Li Z., [38]	China, 2022	RCT	N = 80 patients (40 in the control group and 40 in the observation group); Diagnosed with acute myocardial infarction, age between 30 and 70 years, left ventricular ejection fraction > 50%, good communication skills, and willingness to participate.	Remote Rehabilitation group: Home-based rehabilitation using remote ECG monitoring, guided by medical staff for tailored rehabilitation plans. Control group with traditional rehabilitation. Duration: 6 months	Primary outcomes with improvement in heart function and distance given by the 6 min walking distance. Secondary outcomes: improvement of quality of life, medication compliance, avoid readmissions and adverse events. Significantly improved LVEF and 6MWD compared to the control group at 6 months (421.75 m vs. 346.72 m; *p* < 0.05). Higher medication compliance (92.5% vs. 77.5%; *p* < 0.05) and quality of life scores. Lower rates of unplanned readmissions and adverse cardiac events such as heart failure and unstable angina.	29
11	Sankaran S., [50]	Belgium, 2019	Multidisciplinary Crossover Study	N = 32 patients randomized in 2 groups; only 28 completing the study.; Adult > 18 years with coronary artery disease; history of PCI; clinically stable with no high-risk arrythmias; possession of a smartphone.	HeartHab App Group: Interactive telerehabilitation using the HeartHab app, which provided personalized exercise targets, motivational features, and progress tracking. Control Group: Usual care with no additional digital interventions. Duration: 4 months	Primary Outcomes: Physical activity levels (measured in METs), quality of life (EQ-5D, HeartQoL), and motivation. Secondary Outcomes: Physiological changes (HbA1c, HDL cholesterol), exercise capacity (VO_2_max), and carryover effects post-intervention; Significant improvements in HDL cholesterol (+0.61 mg/dL) and HbA1c (−1.5 mg/dL) during the app usage phase (*p* < 0.05). Increased physical activity (measured in METs) and VO_2_max. Positive carryover effects on motivation, weight, and HDL cholesterol after stopping the app. Enhanced quality of life scores (EQ-5D and HeartQoL). 84% of patients reached or exceeded their prescribed physical activity targets.	32
12	Lee Y.H., [35]	South Korea, 2013,	RCT	N = 55 patients. 26 home-based cardiac rehabilitation, 29 usual care group. Acute coronary syndrome (ACS) with PCI. Age between 18–80 years. Ejection fraction > 30%, Nyha Class I–II, ability to exercise.	Home-based exercise training with wireless monitoring (HeartCall™ device) for 12 weeks. Structured exercise program including scheduled gait exercise (4–5 sessions/week). Gradual increase in exercise intensity monitored via ECG through wireless devices. Regular counseling and feedback via phone. Usual care group with standard medical therapy, diet control, self-managed exercise. Duration: 12 weeks	Primary outcomes: Exercise capacity (METs, exercise time) and quality of life (QOL) scores. Secondary outcomes: rate of perceived exertion. Cardiac rehabilitation group: Significant improvement in METs (+2.47 vs. +1.43; *p* = 0.021), exercise time (+169.68 vs. +88.31 s; *p* = 0.012), and QOL scores (+4.81 vs. +0.89; *p* = 0.022). Greater reductions in submaximal rate pressure product and rate of perceived exertion. Usual care group: Showed modest improvements but significantly less than the CR group.	32
13	Gu J., [48]	China, 2023	Prospective Cohort Study	N = 92 patients (45 in the usual care group, 47 in the remote rehab. group). Coronary artery disease, Age ≤ 75 years, Completion of PCI with a drug-eluting stent within the last 6 weeks, completion of at least 5 weeks of ambulatory cardiac rehabilitation without adverse cardiac events.	Remote Group: Home-based rehabilitation supported by wireless monitoring via Xiaomi MI Band 5, with data collected and analyzed remotely by clinicians. Weekly remote follow-ups were conducted through phone and home visits. Usual care: Traditional hospital-based cardiac rehabilitation sessions, including supervised aerobic and strength exercises, education, and weekly psychotherapy. Duration: 12 weeks	Primary Outcomes: Exercise capacity (6 min walk test (6MWT), VO_2_max) and respiratory anaerobic threshold (VO_2_AT). Secondary Outcomes: Health-related quality of life (HRQoL), anxiety and depression, minor family burden. Both groups showed significant improvement in exercise capacity and HRQoL after 8 and 12 weeks (*p* < 0.05). Remote rehabilitation led to better HRQoL in mental health domains (vitality, role emotional, and mental composite summary scores) compared to in-person CR after 8 weeks (*p* < 0.05). Anxiety and depression scores significantly decreased in both groups, with the remote group showing greater improvement (*p* < 0.05). Family burden scores were significantly lower in the remote group at both 8 and 12 weeks (*p* < 0.05)	32
14	Fang J., [39]	China, 2018	RCT	N = 80 patients 40 in the home-based cardiac telerehabilitation group and 40 in the usual care group. Patient with low-risk post PCI, living with at least one other person, ability to send and receive mobile phone messages. Exclusion criteria: Diabetes, severe comorbidities (e.g., malignancy, severe liver/kidney disease), or cognitive impairment.	Remote Group: Received paper-based CHD booklets and biweekly outpatient reviews educational materials, performed structured walking or jogging thrice weekly, and utilized a remote monitoring system with real-time feedback. UC Group: Received paper-based booklets and biweekly outpatient reviews. Duration: 6 weeks	Primary Outcomes: Exercise capacity (6 min walk test, 6MWT). And improvement of health-related quality of life. Secondary outcomes: Decreased nicotine addiction, depression, and decreased blood pressure. Both groups showed significant improvements in 6MWT, improvement in quality of life, depression end nicotine addiction. The home-based rehabilitation group had significantly greater improvements in exercise capacity (6MWT: +48.2 m vs. +34.77 m; *p* = 0.006) and quality of life (SF-36 physical: +14.18 vs. +6.75, *p* = 0.015; mental: +11.39 vs. +4.27, *p* = 0.021) compared to the usual care group. No significant differences in blood pressure or CDS improvements between groups.	30
15	Campo G., [41]	Italy, 2020	RCT	N = 235 patients (118 in the exercise intervention group, 117 in the control group). Age ≥ 70 years, hospital admission for acute coronary syndrome (ACS), SPPB (Short Physical Performance Battery) score between 4 and 9 at baseline, ejection fraction > 30%, and without valvular diseases.	Exercise Intervention Group: Four supervised sessions (at 1, 2, 3, and 4 months post-discharge) led by a sports physician and nurse. Individualized home-based exercise program including walking and balance exercises performed three times a week. Exercises were based on the Otago Exercise Program and adjusted during each follow-up visit. Control Group: 20 min session of health education on heart healthy lifestyle practice. Duration: 1 year	Improvement in physical performance. Secondary outcomes: quality of life, functional capacity, anxiety and depression; Significant improvement in SPPB scores in the intervention group at 6 and 12 months (baseline: 7.6 to 9.8; *p* < 0.001). Increased grip strength (+16%) and gait speed (+0.18 m/s) in the intervention group compared to the control group (*p* < 0.001). Improved quality of life: EQ-5D scores significantly higher in the intervention group (*p* < 0.001). Lower anxiety and depression scores in the intervention group at 6 and 12 months (*p* = 0.03). Reduction in cardiovascular death and hospitalizations for heart failure in the intervention group (7.5% vs. 17%; *p* = 0.04)	36
16	Lao S. S. W., [34]	China, 2023	RCT	N = 110 patients (experimental group, 55; control group, 55). Post-PCI patients, smartphone ownership, and capability to use mHealth applications.	Experimental group: Cardiac rehabilitation supported by an mHealth application, with components of self-care, exercise monitoring, education, and remote interaction. Usual care with routine follow-up and no mHealth integration. Duration: 6 months	Primary Outcomes: Improvements in physical activity, quality of life, anxiety, and depression levels. Secondary Outcomes: Medication adherence, cardiovascular risk factor modification, exercise self-efficacy, and mHealth utility and satisfaction; Significant reduction in anxiety, depression, total cholesterol, LDL levels, and sedentary time. Improvement in 6 min walk test distance, regular exercise adherence, self-efficacy, and quality of life (*p* < 0.05). The feasibility of mHealth integration was rated satisfactory and effective for supporting cardiac rehabilitation.	34
17	Chen SL, [49]	China, 2024	Prospective Cohort Study	N = 200 patients (100 in the remote management group and 100 in the usual care group). Patients who underwent PCI. Stable coronary artery disease. Ability to use the WeChat app. Willingness to participate in the remote management program	Use of WeChat-based remote management, for proper communication with healthcare professionals, receive educational material on cardiovascular health, receive personalized follow-ups and reminders. The control group received standard care without WeChat.	Improvement of HRQL and adherence to medication, cardiovascular risk factor control, and patient satisfaction. Improvement of HRQOL in WeChat group compared to the control group. Major adherence to prescribed medication and lifestyle modifications in the WeChat group.	32

## Data Availability

The original contributions presented in the study are included in the article, further inquiries can be directed to the corresponding authors.

## Abbreviations

CR	cardiac rehabilitation
PCI	percutaneous coronary intervention
HBCTR	home-based cardiac telerehabilitation
HRQOL	health-related quality of life
6MWT	6 min walking test
CVD	cardiovascular disease
CAD	coronary artery disease
MET	Metabolic Equivalent of Task
HIIT	high-intensity interval training
MICT	moderate-intensity continuous training
